# Second Lieutenant Edward Revere Osler

**Published:** 1917-10

**Authors:** 


**Second Lieutenant Edward Revere Osler,** son of our Associate Editor, died about Sept. 1 of wounds received in service in Belgium, aged 21.

